# Draft Genome Sequence of *Rhodococcus ruber* Strain P25, an Active Polychlorinated Biphenyl Degrader

**DOI:** 10.1128/genomeA.00990-15

**Published:** 2015-09-03

**Authors:** Ekaterina S. Shumkova, Björn E. Olsson, Anna V. Kudryavtseva, Elena G. Plotnikova

**Affiliations:** a A.N. Bach Institute of Biochemistry, Russian Academy of Sciences, Moscow, Russia; b Perm State University, Perm, Russia; c University of Skövde, School of Bioscience, Skövde, Sweden; d Engelhardt Institute of Molecular Biology, Russian Academy of Sciences, Moscow, Russia; e Institute of Ecology and Genetics of Microorganisms, Ural Branch of the Russian Academy of Sciences, Perm, Russia

## Abstract

We report the 5,728,255-bp draft genome sequence of *Rhodococcus ruber* P25, isolated from a soil polluted with halogenated aromatic compounds in the city of Perm, Russia. The strain degrades polychlorinated biphenyls and a broad range of aromatic compounds. It possesses genes that mediate the degradation of biphenyls/polychlorinated biphenyls, naphthalene, and monoaromatic compounds.

## GENOME ANNOUNCEMENT

Actinobacterium *Rhodococcus ruber* P25 (=IEGM896) was isolated from soil polluted with the wastes of a chemical plant producing halogen-containing compounds in Perm (Russia) by enrichment in a liquid minimal medium containing biphenyl. Taxonomic assignment to *R. ruber* P25 was based on a 16S rRNA gene nucleotide sequence (100% similarity with *R. ruber* DSM 43338^T^ [GenBank accession no. X80625]), physiological, and biochemical features analysis (1). Strain P25 was capable of utilizing a broad range of aromatic compounds (biphenyl, phenol, toluene, naphthalene, salicylate, gentisate, *ortho*-phthalate, benzoate, 3-hydroxybenzoate, 4-hydroxybenzoate, and 3,4-dihydroxybenzoate), as well as their substituted derivatives (chlorinated biphenyls and chlorobenzoates, *para*-methylbenzoate, and 2,4-dichlorophenoxyacetate) as a sole carbon and energy source (2–4). *R. ruber* P25 is an active destructor of polychlorinated biphenyls, which are toxic and persistent organic pollutants (5).

To better understand the metabolic versatility of the strain P25, in particular biphenyl and polychlorinated biphenyl destruction pathways, analysis of its genome sequence was carried out. Strain P25 was grown in minimal medium with biphenyl as a sole carbon and energy source. DNA was prepared following a standard genomic DNA purification protocol (6). The draft genome sequence of P25 was prepared using the GS Junior (Roche) system, and the sequencing reads (194,173 reads with an average mean read length of 450 bp) were assembled using the GS De Novo Assembler v2.7. Contigs were ordered using the contig mover function in Mauve (7) using *Rhodococcus pyridinivorans* SB3094 (GenBank accession no. NC_023150.1) as the reference genome. Gene prediction was carried out using GeneMarkS (8). Ribosomal RNA genes were identified using RNAmmer 1.2 (9) and transfer RNAs by ARAGORN (10). Annotation of the predicted protein coding genes was carried out in the Blast2GO software (11).

The draft genome sequence has a total length of 5,728,255 bp and is based on 73 contigs with an *N*_50_ length of 329,488, with the largest contig measuring 483.3 kb. Its analysis showed a G+C content of 70.5%. Contigs constituting 95.6% of the total length (5,479,412 bp) could be ordered by mapping to the reference genome. The draft sequence contains 5,319 coding sequences (CDSs), four rRNAs (5S, 16S, and 23S), and 64 tRNAs genes. The coding regions constitute 91.1% of the total sequence and the average gene length is 964. A total of 3,677 genes (69.6%) were annotated with gene ontology (GO) terms.

Functional annotation showed that strain P25 possesses *bph* genes, benzoate, protokatehoate-, gentisate-, and catechol-degrading genes, and genes of the phenol and naphtalen degradation pathways.

Organization of the *bph* gene cluster differed from other *bph* gene clusters known to date (12–15). It contained genes of the “upper” pathway, transcribed in the same direction: *bphAd-bphD-bphC-bphAa-bphAb-bphAc-bphB* (ferredoxin reductase, 2-hydroxy-6-oxo-6-phenylhexodienoat hydrolase and 2,3-dihydroxybiphenyl 1,2-dioxygenase*,* biphenyl 2,3-dioxygenase α- and β-subunites, ferredoxin, biphenyl 2,3-dihydrodiol dehydrogenase, respectively). This order is not typical. The genes of the “lower” pathway of biphenyl degradation, encoding 4-hydroxy-2-oksovalerat aldolase, acetaldehyde dehydrogenase, and 2-keto-4-pentenoate hydratase, are located downstream of *bphB* and are transcribed in the opposite direction.

### Nucleotide sequence accession numbers.

This whole-genome shotgun project has been deposited at DDBJ/EMBL/GenBank under the accession no. LDUF00000000. The version described in this paper is version LDUF01000000.
